# Analysis of DME/DME Navigation Performance and Ground Network Using Stretched-Front-Leg Pulse-Based DME

**DOI:** 10.3390/s18103275

**Published:** 2018-09-29

**Authors:** Euiho Kim

**Affiliations:** Department of Mechanical & System Design Engineering, Hongik University, 04066 Seoul, Korea; euihokim@hongik.ac.kr

**Keywords:** DME, GNSS jamming, APNT

## Abstract

Global navigation satellite systems (GNSS) have become a primary navigation means for aircraft. However, the signal power of GNSS is very weak, and its service can be disrupted at any time when there is interference or jamming. For this reason, the Federal Aviation Administration (FAA) in the United States has recently chosen a distance measuring equipment (DME)-based aircraft navigation technique, called DME/DME, as an alternative aircraft navigation means for use by around 2030. The reason that the FAA plans to use DME/DME in such a short duration, by around 2030, is presumed to be because the ranging accuracy of DMEs is between 70 to 300 m, which is about 7 to 30 times worse than that of GNSS. Thus, a significant loss of positioning performance is unavoidable with current DMEs. To make DME/DME a more competent alternative positioning source, this paper proposes an advanced DME that could provide a ranging accuracy of around 30 m by employing a recently developed Stretched-Front-Leg (SFOL) pulse. The paper introduces optimal ground station augmentation algorithms that help to efficiently transform the current DME ground network to enable a DME/DME positioning accuracy of up to 0.3 nm or 92.6 m with a minimal number of new ground DME sites. The positioning performance and augmented ground network using the proposed SFOL pulse-based DME are evaluated in two regions which have distinct terrain conditions.

## 1. Introduction

Global navigation satellite systems (GNSS) have been widely used for aircraft navigation, and their role will be more important in future air traffic control. For example, the USA Next Generation Air Transportation System (NextGen) and Single European Sky ATM Research (SESAR) in Europe heavily depend on GNSS to meet future air traffic demands. However, it is well known that the GNSS signal can be easily lost in the presence of interference and jamming because of its weak signal power. In fact, South Korea experienced Global Positioning System (GPS) outages in 2010 and 2012 due to the strong GPS jamming signal transmitted from North Korea [1]. These events caused serious safety issues for aircraft and ships using GPS as a primary navigation source.

In fact, GNSS outages due to interference or jamming were expected before the events in South Korea. The Federal Aviation and Administration (FAA) in the USA started an Alternative Position, Navigation, and Timing (APNT) program in 2010 to develop a GNSS back-up system for possible GNSS outages [2,3]. The envisioned APNT system by the FAA should have resilience such that it is difficult to interfere with. In addition, it must be able to continuously operate without being dependent on GNSS, and it should be able to serve various users such as commercial airliners, as well as general aviation. Another important APNT system requirement is that its service should be compatible with the legacy users, as well as future users such as unmanned aircraft systems.

Many clever approaches have been proposed for an APNT architecture, including wide area multilateration (WAM), GNSS-like pseudolites, distance measuring equipment-based navigation (DME/DME) [4,5], enhanced DME using a carrier phase [6], high-accuracy DME using alternative pulses [7,8], a mosaic DME/pseudolite hybrid system [9], and L-band Digital Aeronautical Communication System type 1 (LDACS1) [10]. First, WAM uses aircraft surveillance signals such as Automatic Dependent Surveillance-Broadcast or 1090 Extended Squitter as ranging sources. Using the known location of WAM receivers and their range measurements, a WAM master station computes an aircraft position and sends the position solution to the aircraft. Because of the delivery of the aircraft position from the ground, there has been concern regarding the security of the system. The GNSS-like pseudolites operate similarly to GNSS with much higher signal power, except that ranging sources are installed on the ground rather than in outer space. This approach requires a pseudolite network infrastructure with a precise time synchronization, which requires significant resources. The DME/DME is a traditional ground nav-aid system, and a DME ground network is well established around the world. In DME/DME positioning, the aircraft measures a slant range to the ground DME transponders in view and computes its position through the multilateration technique. Because the aircraft position is determined onboard, there is no security concern like with WAM. However, the drawback of the DME/DME is a poor positioning accuracy, which can be a few hundred meters. To improve DME/DME positioning accuracy, the enhanced DME was proposed. This method uses a DME carrier phase to measure the distance between aircraft and a ground DME transponder in centimeter levels of accuracy, which requires a more precise oscillator than the one used in today’s DME system. On the other hand, the proposed alternative pulses are to improve DME/DME positioning accuracy by replacing the conventional Gaussian pulse. The alternative pulses are able to suppress multipath and noise impact on the range measurements. It seems to be possible to transmit the alternative pulses in a modern existing DME without a hardware change but a power amplifier pre-distortioner algorithms tuned for a Gaussian pulse should be adjusted for an alternative pulse. The mosaic DME/pseudolite hybrid system proposed to use one DME ground station with a few pseudolites. This unique design enables an aircraft 3D positioning with one DME station, but its positioning accuracy can be up to several hundred meters, and a pseudolite installation is required. The LDACS1 is a future aeronautical communication system in L1 band and can be used as a ranging source, as well. This system has been actively investigated and requires further research at this point.

Among these candidates, the FAA has recently chosen DME/DME as a short-term APNT solution by 2030, and has planned to augment the currently deployed DME ground transponder network to fill DME/DME coverage gaps in the conterminous USA [11]. It is presumed that the reason for using DME/DME as an APNT is the well-established DME ground network and widespread use of DME avionics in commercial airliners. DME measures the slant range between the aircraft and the ground transponder based on the radar principle [4]. Using the multiple ranges from ground DME transponders, the DME/DME positioning technique computes the aircraft position using multilateration. One of the drawbacks of using the legacy DME equipment for APNT is the lack of ranging accuracy. The ranging accuracy of today’s DME is around 7 to 30 times worse than GNSS ranging accuracy [5], which is insufficient to support the targeted APNT positioning accuracy for navigation (0.3 nm) and surveillance (92.6 m) originally proposed in [4,5]. To improve DME raging accuracy, alternative pulse waveforms have been proposed [7,8], including smoothed concave polygon (SCP) and stretched-front-leg (SFOL) pulses. A recent analysis of the SFOL pulse showed that the multipath-induced ranging error of the SFOL pulse was about four and half times and two and half times less than the conventional Gaussian and SCP pulses, respectively. Therefore, if an SFOL pulse is implemented in DMEs, its higher ranging accuracy could significantly improve DME/DME positioning accuracy.

This paper assesses a possible ranging accuracy of an SFOL pulse-based DME and investigates DME ground network augmentations to meet a DME/DME positioning accuracy of 0.3 nm or 92.6 m in two distinct terrain conditions. The augmentation of a DME ground network is planned in a way that a minimal number of new DME sites is added to the existing DME network while meeting the desired alternative aircraft positioning accuracy. In this paper, Section 2 briefly gives an overview of the performance of today’s DME and desirable APNT performance requirements. Section 3 introduces the proposed SFOL pulse-based DME. Section 4 presents DME ground network optimization algorithms based on binary integer linear programming. Section 5 presents the optimized DME ground networks used to support a horizontal positioning accuracy of 0.3 nm and 92.6 m in selected regions. A discussion of the results follows in Section 5.

## 2. Overview of Distance Measuring Equipment and Requirements on Alternative Aircraft Positioning

DME consists of ground transponders and aircraft avionics. Aircraft DME avionics first interrogate ground transponders, and the transponder replies back to avionics through a pair of pulses. The time elapsed from the interrogation to the receipt of the replies is used to measure the slant range between the aircraft and the ground transponders [4,11]. The operational frequency ranges of the DME are from 960 to 1215 MHz. This frequency range is divided into 252 channels, and each channel is separated by 1 MHz, and a ground DME transponder is assigned its own channel frequency. To find the aircraft position using DMEs, referred to as DME/DME positioning, multi-channel or scanning DME avionics simultaneously measure slant ranges to multiple ground transponders in view and compute aircraft position using the known coordinates of the transponders stored in a flight computer and multilateration principles. The number of available channels in a scanning DME is typically between two and six.

To use DME/DME as an alternative positioning means to GNSS, its service area should be down to terminal area where aircraft positioning accuracies satisfy 1 nm (95%) [12]. It would also be desirable for DME/DME to be capable of supporting an approach phase of flight, which requires up to 0.3 nm positioning accuracy. In addition, to use DME/DME as a surveillance system, its positioning accuracy should be better than 92.6 m, which is the aircraft tracking accuracy of secondary surveillance radars (SSR) [5].

The accuracy requirement for DME/DME-based navigation, i.e., total system error (TSE), is a statistical sum of navigation system error (NSE), flight technical error (FTE), and path definition error (PDE) [3], such that

(1) $$TSE^{2}=FTE^{2}+NSE^{2}+PDE^{2}\;$$

The NSE is also called a position estimation error, and FTE is a path steering error and depends on the flight control method. The PDE is the error associated with route waypoints and is usually considered to be zero, because waypoints can be accurately surveyed nowadays. The NSE is caused by a positioning system and can be formulated for DME/DME as follows

(2) $$NSE=HDOP\cdot \sigma_{range}\;$$

where *HDOP* is a horizontal dilution of precision, and $\sigma_{range}^{}$ is the ranging accuracy of DME. *HDOP* is a goodness of the relative geometry between the aircraft and the ground DME transponders in view and can be computed from a positioning error covariance matrix. For a DME positioning system to meet an NSE, the required *HDOP* is given by

(3) $$HDOP=\sqrt{\frac{TSE^{2}-FTE^{2}}{4\sigma_{range}^{2}}}\;$$

For example, an FTE of 0.25 nm (95%) and $\sigma_{range}^{}$ of 70 m (95%) require a 4.39 or lower *HDOP* value to satisfy a TSE value of 0.3 nm. Please note that *HDOP* depends on a ground network layout, and the ground network optimization algorithms discussed in Section 4 search for an efficient ground network layout that provides a targeted *HDOP*. 

## 3. SFOL Pulse and Proposed SFOL-Based DME

The conventional DME widely uses Gaussian pulses. The Gaussian DME pulse has a narrow spectral density that does not interfere with other neighborhood channels, but its multipath and noise-induced range error could be large, up to a hundred meters, due to its slowly increasing rising edges. This section introduces an alternative SFOL DME pulse that is insensitive to multipath effects and significantly enhances a DME ranging accuracy. This section also presents an advanced DME that is based on the SFOL pulse and DME/P (precision) like hardware.

### 3.1. Overview of SFOL Pulse

**Figure 1:**
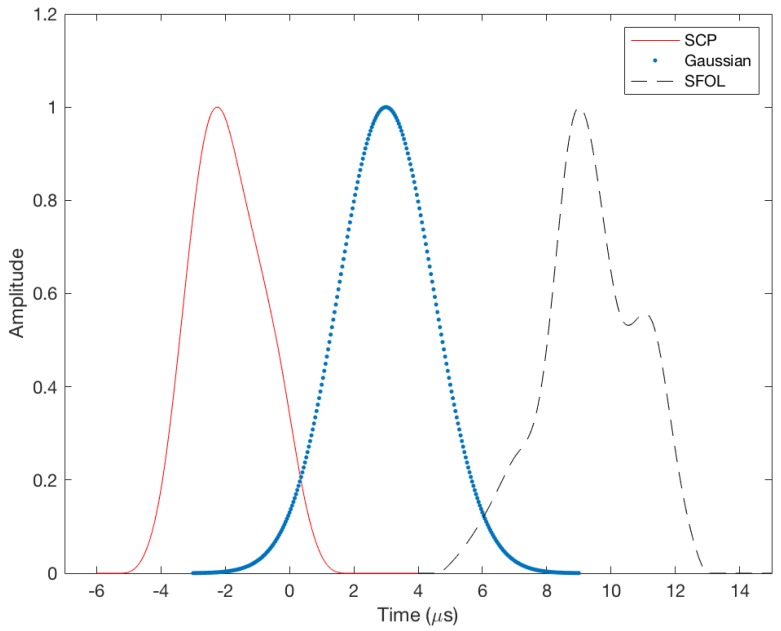
Comparison of Gaussian, Smoothed Concave Polygon, and Stretched-Front-Leg pulses.

The SFOL pulse was designed by using genetic algorithms [8]. The SFOL pulse has a rise time of 2.8 μs, a width of 3.4 μs, and a falling time of 3.0 μs. Thus, it satisfies the pulse shape requirements in the current DME ground transponder specifications [13]. Figure 1 compares a conventional Gaussian pulse, SCP pulse, and SFOL pulse. The overall shape of the SFOL pulse is very different from the other two pulses, and the unique shape of the rising edge is effective in reducing multipath effects.

**Figure 2:**
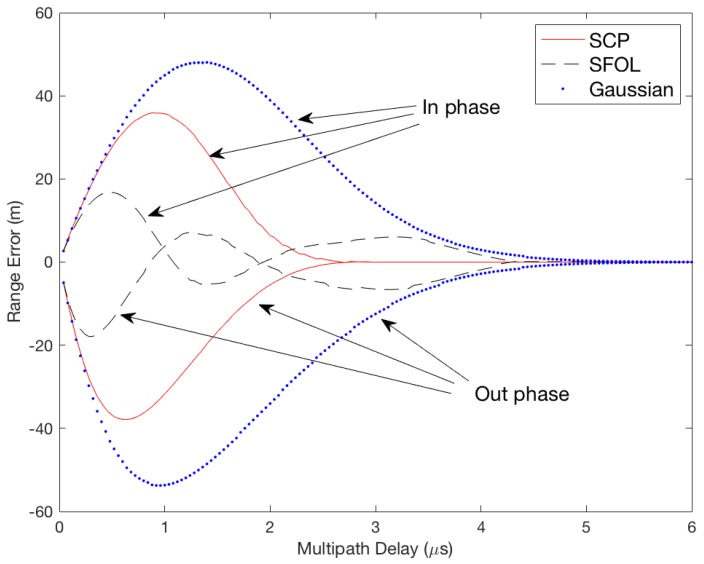
Range error envelopes due to in-phase and out-of-phase multipaths for SCP, SFOL, and Gaussian pulses.

**Figure 3:**
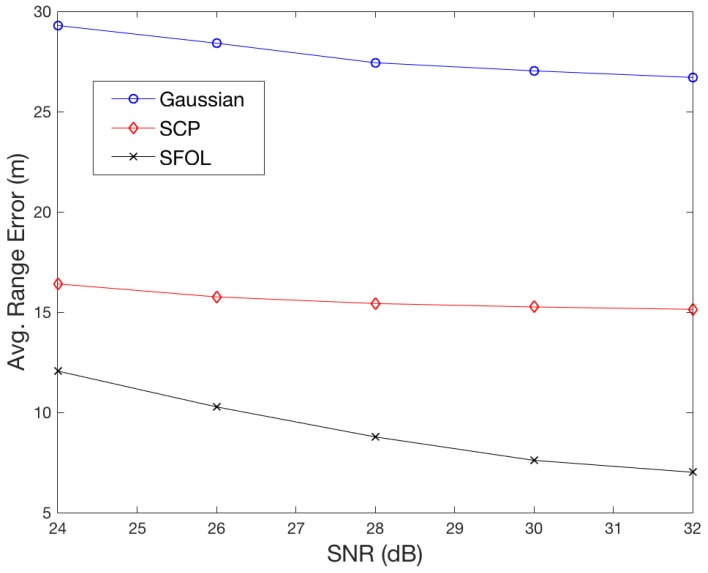
Averaged root mean square (RMS) range errors in 1000 cases of noise injection with five levels of signal-to-noise ratio (SNR).

Figure 2 compares the ranging performance of the Gaussian, SCP, and SFOL pulses with respect to the injected multipath. Multipath delays range from 0 to 6 μs, and the multipath to direct pulse amplitude ratio is 0.3. With the injected multipath conditions, the root mean square (RMS) of the multipath-induced ranging errors of Gaussian and SCP pulses were 26.1 and 14.9 m, respectively. The RMS of SFOL was 5.9 m. Figure 3 shows the sensitivity of the multipath mitigation performance of the SFOL pulse under a noisy environment. As the signal-to-noise ratio decreases, the multipath mitigation performance of the SFOL pulse tends to decrease as well. However, the SFOL pulse is still able to mitigate multipath range errors by 56.9% and 36.0% of those of Gaussian and SCP pulses, respectively, even at an SNR of 24 dB.

### 3.2. Proposed SFOL Pulse-Based DME

The primary range error sources in DME include timing biases, multipath, and thermal noise. The ranging error budgets in the DME specifications and modern state-of-the-art DMEs are listed in Table 1 [5]. It is also expected that current DMEs being operated are the mixture of the legacy DMEs barely meeting specifications and modern state-of-the-art DMEs. Although the SFOL pulse could effectively reduce multipath-induced ranging errors, suppression of other ranging error sources is also desirable to have a better ranging accuracy, as shown in Table 1.

To maximize DME ranging accuracy with current technologies, it is recommended to transmit an SFOL pulse in precision DME (DME/P)-like hardware that provides 10–30 m ranging accuracy depending on operational modes [14]. DME/P was originally developed to support aircraft automatic landing with a microwave landing system. Because DME/P uses a cos/cos^2^ pulse shape with an abrupt change at the rising edge, which causes a wide spectrum, its transmission power is typically less than 100 Watts to avoid being an interference source to other nav-aid signals. Therefore, the service coverage of DME/P is at most 20 nm or so. If an SFOL pulse is employed in DME/P hardware, referred to DME/S (DME/SFOL) in this paper, DME/S would be able to provide high-ranging accuracy with the same coverage area as DME/N (DME Normal) up to approximately 200 nm without any interference concerns. The projected ranging accuracy budget of DME/S is also listed in Table 1. The timing biases in the transponder and avionics were taken from DME/P standards [14]. The ranging accuracy of propagation and noise was based on simulation results that the SFOL pulse mitigated multipath and noise-induced errors by 56.9% at 24 dB SNR, as shown in Figure 3.

## 4. DME Ground Network Development Strategy

The current DME ground network is mainly the legacy of DME and Very High Frequency omnidirectional range (VOR)-based navigation, called rho-theta navigation. In rho-theta navigation, the aircraft needs to see one DME collocated with VOR, while DME/DME positioning must have lines of sight to two or more ground DME stations. Thus, DME/DME requires a greater number of ground stations than rho-theta navigation, and the FAA is currently augmenting the DME ground network to fill the DME/DME coverage gaps in the conterminous USA [12]. This section discusses how a DME ground network can effectively augment the existing DME network using binary integer linear programming [15,16].

### 4.1. Overall Architecture Development Approach

**Figure 4:**
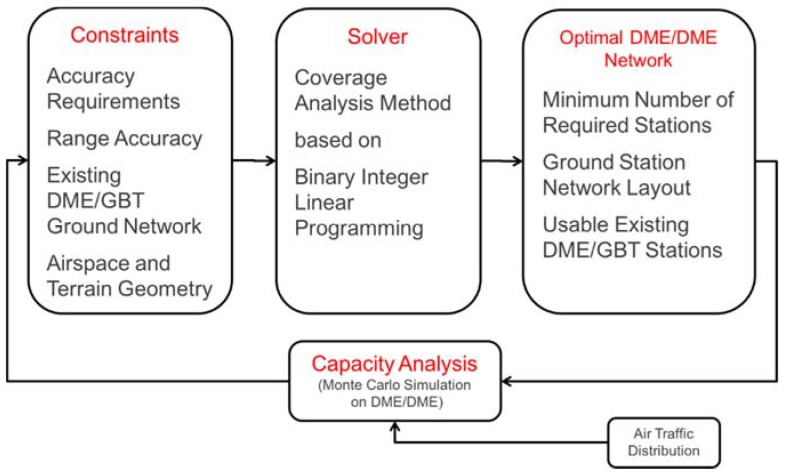
DME/DME network placement procedures.

The optimization of a DME/DME ground network is determined through the process shown in Figure 4. The process takes the following parameters as input: TSE, FTE, existing DME station network topology, defined airspace, terrain geography, and $\sigma_{range}^{}$. Using these inputs, the process involves searching for an optimal ground station network having a minimum total number of stations while possessing the maximum number of existing DME stations. Because DME/DME operation is capacity-limited, it is necessary to perform a capacity analysis to check if the determined optimal station layout can handle the interrogation requests from the expected future air traffic. In the previous analysis, it was found that a ground network meeting the positioning accuracy requirement had no problem in processing the interrogation rates of future air traffic [15]. Therefore, the capacity analysis is not considered in this paper.

#### Coverage Analysis Methodology Using Binary Linear Integer Programming

The proposed process generates an optimized DME/DME ground station network by filling the coverage gaps of the currently deployed DME ground network to provide the required alternative positioning service. When there are only small local coverage gaps, the augmentation problem is straightforward. However, when the coverage gaps extend to a large area, augmenting new DME sites to an existing ground network becomes a complex sensor network design problem.

These facility location or sensor network problems have been an active research subject for several decades [17]. The sensor network problem is nondeterministic polynomial time (NP)-complete; therefore, heuristics-based search algorithms tailored for a particular problem have to be used, because there are no general efficient algorithms [18]. For example, Roa et al. developed a meta-heuristic approach which balanced the precision and availability of positioning and looked for a minimum number of ranging sources through a diversified local search [19]. Genetic algorithms have been used to design an optimal sensor network using dilution of precision (DOP) or its variant as a fitness function [20,21]. Ref. [22,23] optimized the internode distances of ranging sources to find a positioning network resulting in a minimum average mean square error (MSE) in the estimated user position. These prior studies looked for optimal sensor layouts in a limited space with few constraints. The optimal planning of augmenting DME ground networks must consider a much larger area and constraints such as existing DME sites, terrain geometry, radio propagation, and airport location. This section presents heuristics-based sensor planning algorithms that are tuned for the DME augmentation problem.

**Figure 5:**
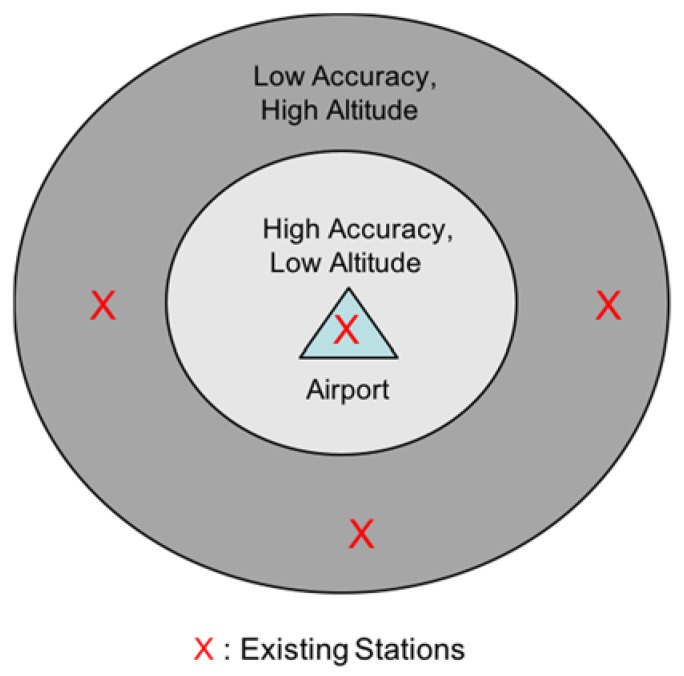
Station placement heuristics set-up.

To develop effective heuristics for the DME augmentation problem, it is necessary to understand the characteristics of DME/DME-based aircraft navigation. First, as shown in Figure 5, an aircraft requires more stringent positioning accuracy, as its altitude is lower around an airport. However, in the lower altitudes, the line of sight from aircraft to a DME ground becomes limited such that positioning accuracy is likely lower than in high-altitude airspace. Second, the lower *HDOP* induces better positioning accuracy, and lower *HDOP* can generally be a result when a user is surrounded by ranging sources. Aircraft at high altitudes do not usually play a role in the design of a DME network because it can see many DMEs with a good azimuth angular diversity and can easily meet the required accuracy [15]. Therefore, useful heuristics include first placing DMEs outside low-altitude regions such that aircraft meet a more stringent positioning requirement. Also, DMEs on the ground must have some distance between them to avoid placing multiple DMEs in a local area, which may result in redundant sites. An additional heuristic is the preference for existing stations over new stations because the re-use of the existing stations would be expected to reduce the overall APNT architecture development costs.

With those heuristics, the method proposed below uses a two-step binary integer linear programming (BILP) formulation to search for valid solution sets. In this approach, terrain, and airspace were sectored into grids. The formulation of the first step is

(4) $$\begin{array}{l} \min Z=\sum_{i=1}^{n}w_{i}x_{i}=w^{T}x\\ \mathrm{subject}\;\mathrm{to}:\;Ax\geq b\\ \;\;\;\;\;\;Cx\leq d\\ \;\;\;\;\;\;Fx\leq g\\ \;\;\;\;\;\;w^{T}x\leq Z_{\min}\\ \;\;\;\;\;\;x_{i}\in \left\{0,1\right\} \end{array}$$

where *Z* is the cost function of a candidate DME network. *x* is the index vector of a candidate station location. If *x_i_* = 1, then it contains a candidate station. Otherwise, *x_i_* = 0. The vector **w** is a weighting factor on a particular station location, and its element value is lower at the preferred candidate station locations. The matrix **A** is a visibility matrix and has information about the radio propagation line of sight between aircraft in a given location and the candidate station locations. The *i*th row of **A** corresponds to an aircraft location grid and the *j*th column to a candidate station location grid. The elements of matrix **A** also take on values of 0 or 1. If the aircraft at the *i*th row location “sees” the station at the *j*th column location, *A_i,j_* is set to 1. Otherwise, *A_i,j_* is 0. The vector **b** represents the required minimum number of visible stations at the corresponding aircraft location.

**C** and **d** control the minimum separation between stations. If the distance between *i*th and *j*th stations is less than the minimum separation, *C_i,j_* is set to 1. Otherwise, *C_i,j_* is equal to 0. The vector **d** is a vector of all 1s, which means that only one station is allowed to be inside the separation limit of another station location. The matrix **F** contains records of the previous solution sets **x**, and the vector $g=(x^{T}1-1)1$ has all of its values equal to one less than the number of stations in the previous solution sets in **F**. This constraint forces the BILP formulation to yield a unique solution for each iteration. Finally, *Z*_min_ is the minimum cost among the valid solution sets found through previous iterations. Thus, the last constraint, **w***^T^***x** ≤ *Z*_min_, reduces the search space such that the BILP only looks for a new solution having a cost that is less than or equal to *Z*_min_. 

Please note that Equation (4) does not have the accuracy requirement as a constraint, because it cannot be directly incorporated into the formulation. Therefore, the solutions from Equation (4) do not necessarily satisfy the accuracy requirement. For this case, the coverage analysis method implements the second step or updated BILP in Equation (5).

(5) $$\begin{array}{l} \min Z=\sum_{i=1}^{n}w_{i}x_{i}=w^{T}x\\ \mathrm{subject}\;\mathrm{to}:\;Ax\geq b_{\mathrm{mod}}\\ \;\;\;\;\;\;C_{\mathrm{mod}}x\leq d\\ \;\;\;\;\;\;F_{\mathrm{mod}}x\leq g\\ \;\;\;\;\;\;w^{T}x\leq Z_{\min}\\ \;\;\;\;\;\;x_{i}\in \left\{0,1\right\} \end{array}$$

where $b_{\mathrm{mod},i}=\{\begin{array}{l} b_{i}+1\;\mathrm{if}\;HDOP_{i}>HDOP_{\mathrm{req}}\\ b_{i}\;\mathrm{if}\;HDOP_{i}\leq HDOP_{\mathrm{req}} \end{array}$.

This second step of Equation (5) is repeated until a solution set meeting the required *HDOP* is found or no solution is found due to the conflicts with the constraints. The impact of the vector **b**_mod_ is that the airspace locations with an insufficient *HDOP* value need visibility to at least one more station to improve the *HDOP* value. The matrix **C**_mod_ relaxes the separation between stations at small steps for each iteration, and **F**_mod_ is used only for the updated BILP formulation. The BILP may fail to find any solutions that meet the constraint in the beginning. This usually happens when the minimum separation in **C** is too conservative, such that the possible combinations of the valid ground stations do not exist. In that case, it is necessary to relax the separation constraint between stations to search for valid solution sets.

**Figure 6:**
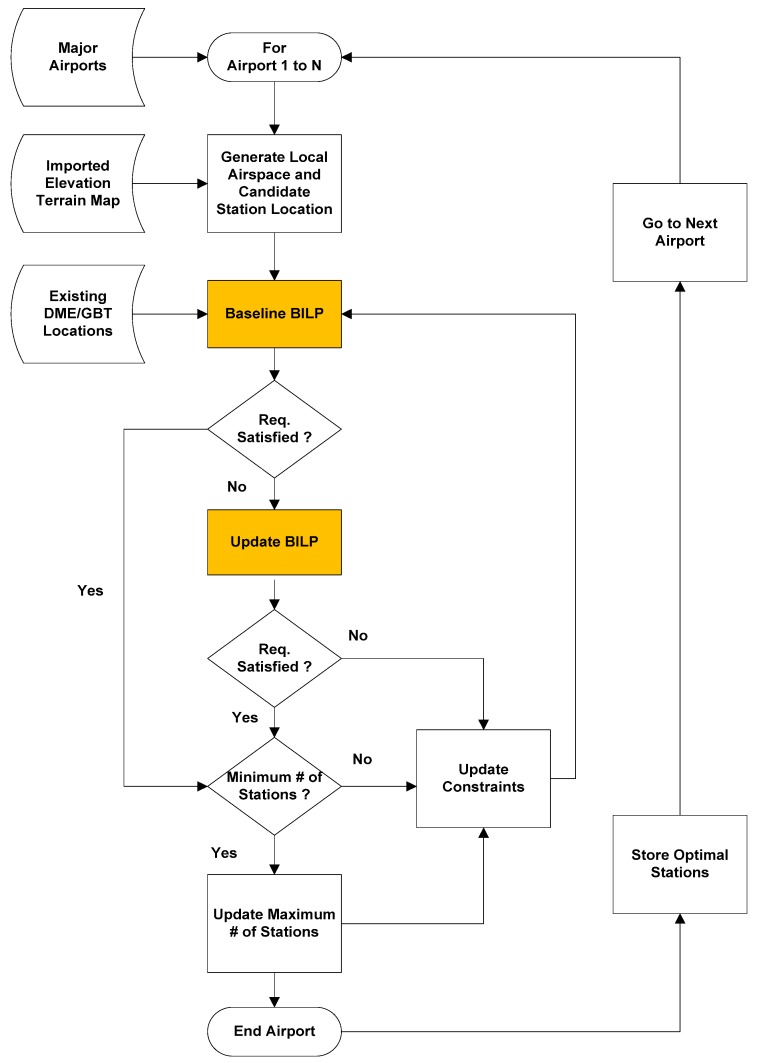
Overall coverage analysis methodology process.

The block diagram in Figure 6 shows the proposed overall method in designing an optimal DME ground station network using Equations (4) and (5). As shown in the process, the BILP does not try to find a ground network for the airspace in the entire interested region at once because the search space that BILP has to handle can be typically extremely large. Instead, BILP searches for an optimal ground network for each airport and check if there are any coverage gaps in the entire airspace after finishing the search procedure for all of the airports. This approach not only accelerates the search space by reducing the dimension of the vectors and matrices in Equations (4) and (5), but also finds a ground network that provides a complete coverage of the entire region. The reason for this is that a low-altitude airspace with a limited line of sight to the ground DME transponders only exists near an airport. With the ground network layout satisfying the low altitude, aircraft at high altitudes of over 5000 ft have no problem in meeting the position accuracy requirements since there are usually several ground transponders in view at high altitude.

For the preparation of the ground network search around an airport, the BILP first generates grids of the airspace and terrain. Using the coordinates of the grids, the vector **x** and the matrices **A** and **C** are generated by using a digital terrain map and a Matlab radio propagation analysis tool. The vector **b** can be set to a column vector of 2s, which is the minimum number of a ranging source required to compute a horizontal position. Please note that the matrices **A** and **C** and the vector **b** in the first step should be conservatively set to avoid a redundant site. While the first step is implemented once, the second step is iterated multiple times until every airspace grid meets the required positioning accuracy. At each iteration of the second step, **C**_mod_ allows a smaller distance between ground transponders and the element value of the vector **b**_mod_ increases by one such that the airspace grid having an insufficient positioning accuracy would be able to see an additional ground transponder. The process in Figure 6 tends to result in a minimum number of required ground transponders, and its optimality was rigorously discussed in [15]. This process is used to generate optimized DME/DME networks for the selected regions in the next section.

## 5. Optimized DME/DME Network with DME/S in Two Selected Regions

In this section, the optimized DME/DME network with the proposed DME/S is investigated using the coverage analysis procedure for the following two selected regions: South Florida and South Korea [24,25]. The two regions have very different terrain conditions. In South Florida, most terrain is flat, such that an aircraft has no problem in having lines of sight to multiple DMEs. However, the terrain in South Korea consists of numerous high mountains that significantly limit the lines of sight between aircraft and ground transponders. These cases studies will shed light on appropriate APNT positioning performance requirements based on DME/DME with DME/S in different regions. In the studies, aircraft are assumed to be equipped with a three-channel scanning DME for DME/DME positioning. When more than three DME ground transponders are in view of the aircraft, it is also assumed that the scanning DMEs can select a set of ground transponders that minimize the *HDOP* value. For the evaluation of the coverage, the airspace was sampled into grids 1 km apart in longitudinal and lateral directions. The altitudes of the grids were determined from the airspace definition in [2] and the line of sight information between aircraft and a ground transponder was provided from Matlab radio signal propagation software. The coverage of the airspace was determined if the computed aircraft horizontal position accuracy, i.e., the product of *HDOP* and the assumed DME ranging accuracy meets a given positioning accuracy requirement.

### 5.1. Case Study: South Florida

**Figure 7:**
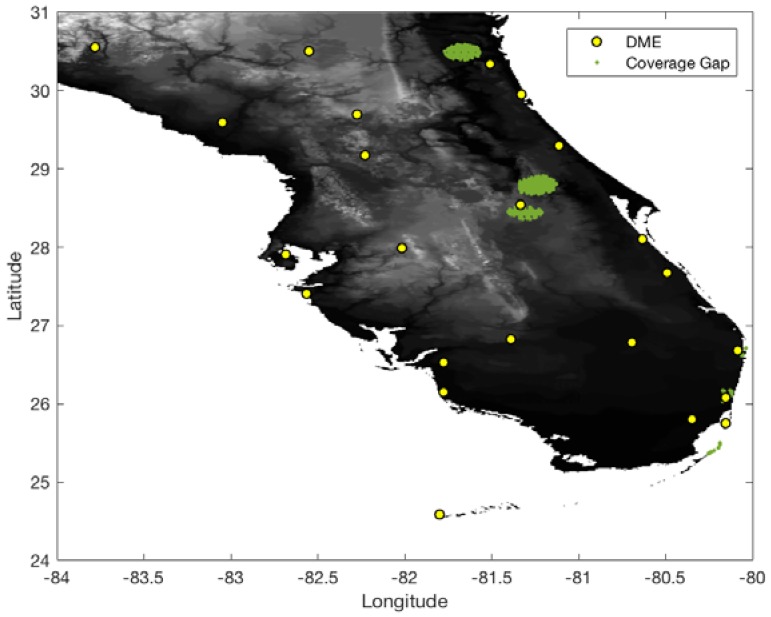
DME/DME positioning accuracy gaps of 1.0 nm with legacy DME and the current DME ground network.

**Figure 8:**
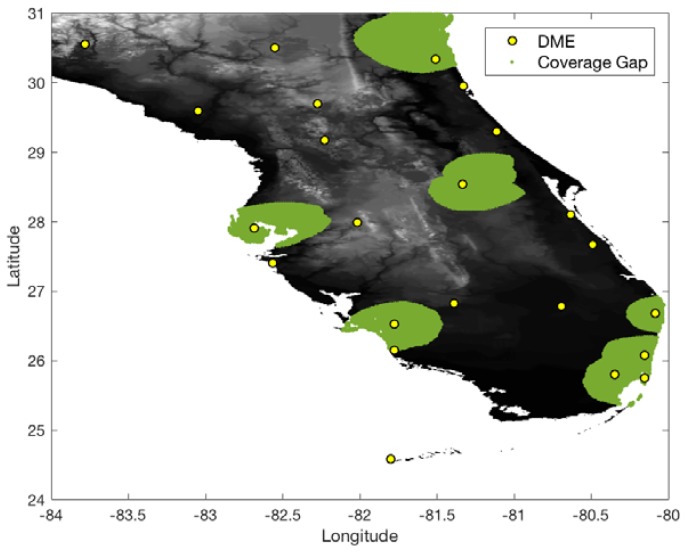
DME/DME positioning accuracy gaps of 0.3 nm with legacy DME and the current DME ground network.

In the South Florida region, there are eight international airports and 23 DME sites. The airspace altitude defined in this work is at 500 ft above ground level (AGL) within a 5 statute mile (SM) radius of an airport and gradually increases up to 5500 ft [2]. To conservatively evaluate the DME/DME coverage of the current ground network in the South Florida region, the ranging accuracy of legacy DME is set to 370.4 m (95%) (Table 1). With the ranging accuracy and current ground DME network, Figure 7 and Figure 8 show 1.0 and 0.3 nm positioning accuracy coverage gaps, respectively. The positioning gap of 1.0 nm is local and could easily be removed by adding a couple of DMEs near the gaps. However, the positioning gap of 0.3 nm is large, and a significant number of additional DMEs would be needed to provide 0.3 nm positioning capability.

**Figure 9:**
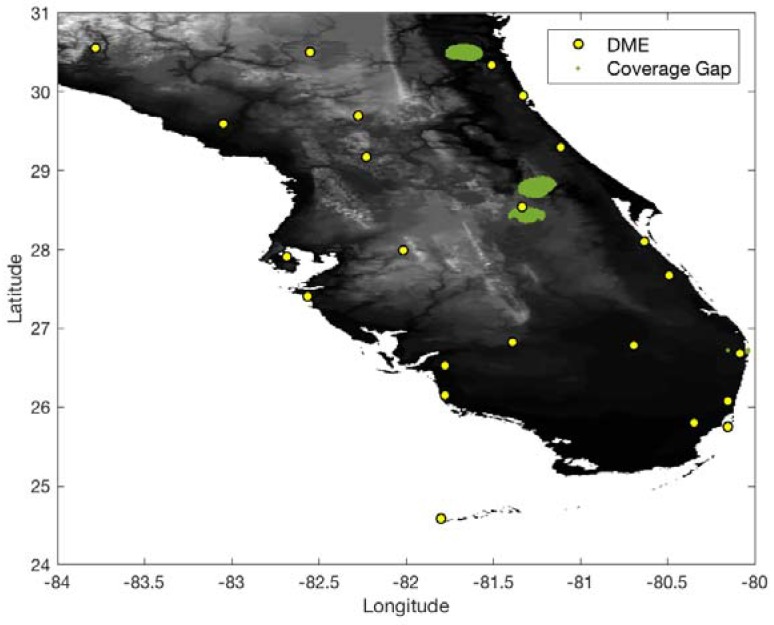
DME/DME positioning accuracy gaps of 0.3 nm with DME/S and the current DME ground network.

**Figure 10:**
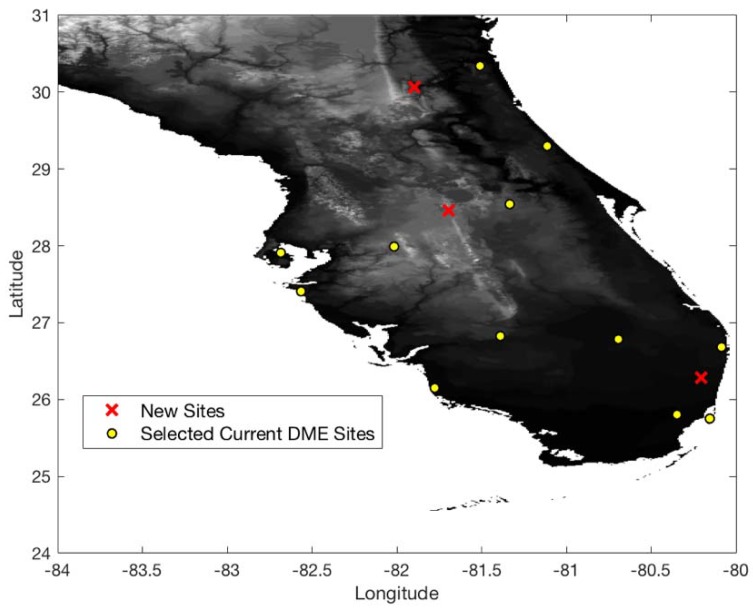
Optimal DME transponder network for 0.3 nm positioning accuracy with DME/S.

**Figure 11:**
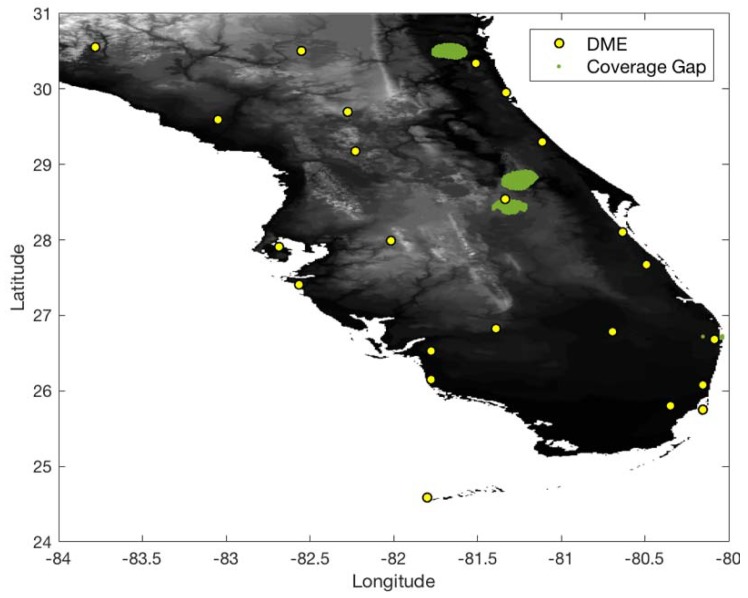
DME/DME positioning accuracy gaps of 92.6 m with DME/S and the current DME ground network.

**Figure 12:**
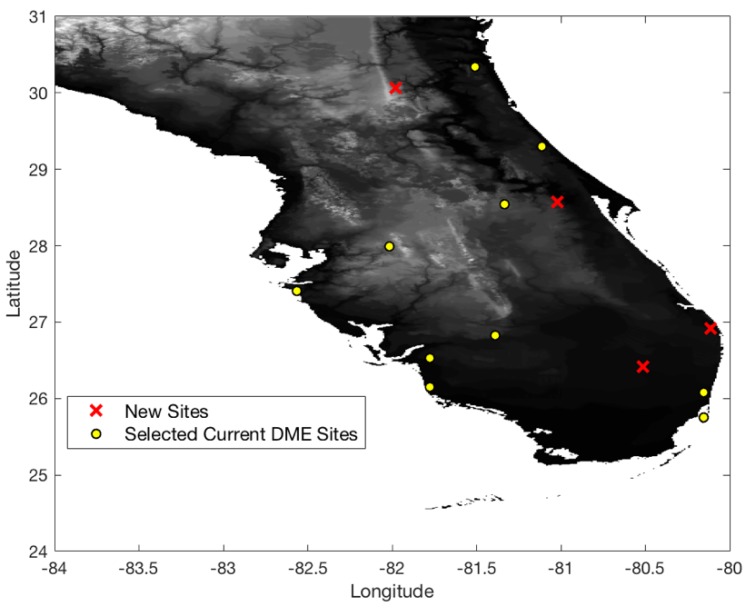
Optimal DME transponder network for 92.6 m positioning accuracy with DME/S.

Figure 9 shows 0.3 nm positioning accuracy coverage gaps when DME/S and the current ground DME network are used, which eliminates most coverage gaps of the legacy DME except for some small low-altitude regions near airports. Figure 10 shows the optimized ground network of DME/S, which provides 0.3 nm positioning accuracy in the entire region. The network in Figure 10 required a total of 15 DME sites, with three new sites, and could eliminate 11 current DME sites. Figure 11 shows the positioning accuracy of coverage gaps of 92.6 m with DME/S and the current ground DME network. Please note that the coverage gap of 92.6 m positioning accuracy is almost identical to that of 0.3 nm positioning accuracy. Figure 12 shows the DME/S ground network for 92.6 m positioning accuracy. This network requires a total 14 of DME sites with four new sites.

### 5.2. Case Study: South Korea

In the South Florida case, a DME/DME horizontal positioning accuracy of 1.0 nm or 0.3 nm could be achieved using DME/S with a few new sites. The DME/DME positioning accuracy of 1.0 nm is met in most areas with the current network of the legacy DME. However, the difficult terrain conditions in South Korea make DME/DME positioning more complicated. 

**Figure 13:**
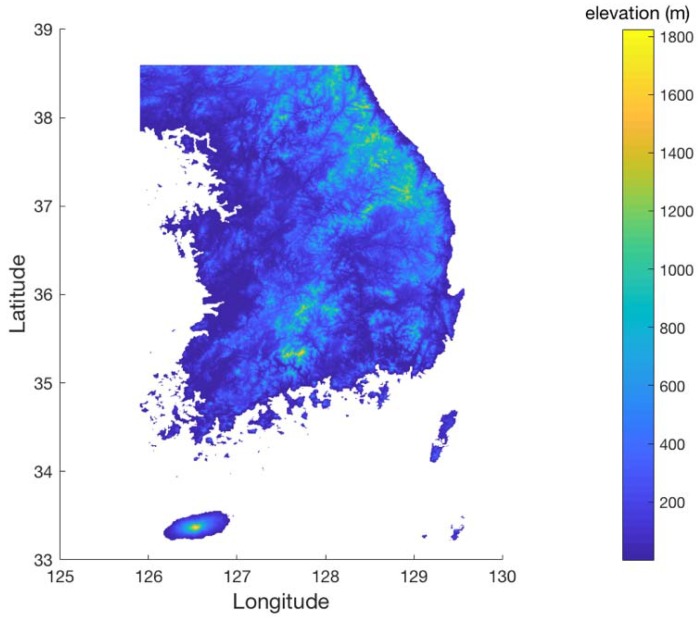
Terrain elevation profile in South Korea.

**Figure 14:**
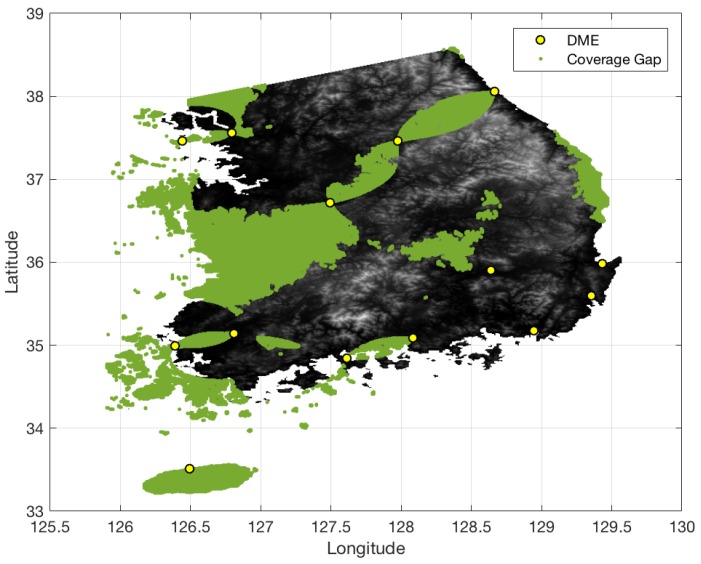
DME/DME coverage of 1.0 nm horizontal positioning accuracy in South Korea with legacy DME performance and the current DME ground network.

**Figure 15:**
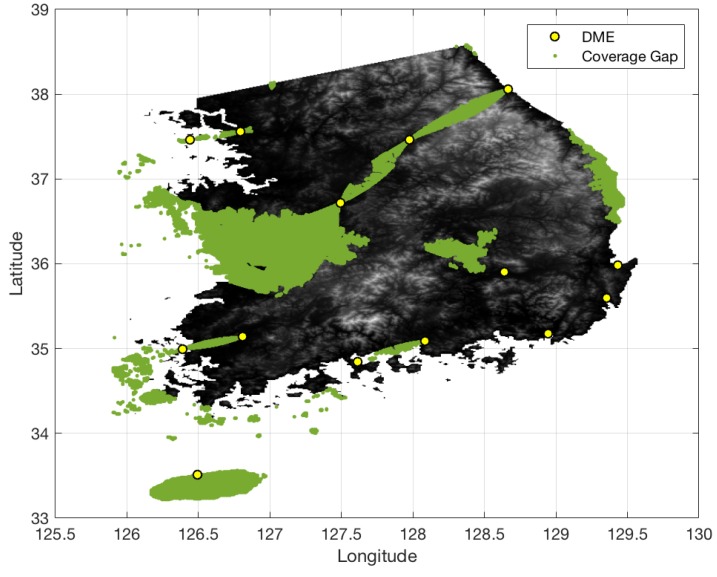
DME/DME coverage of 0.3 nm horizontal positioning accuracy in South Korea with DME/S and the current DME ground network.

**Figure 16:**
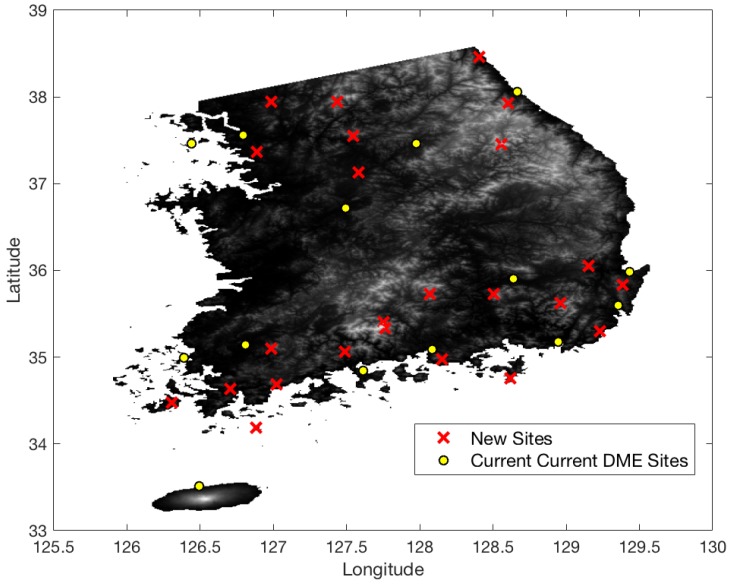
Augmented DME network for 0.3 nm horizontal positioning accuracy with DME/S in South Korea.

**Figure 17:**
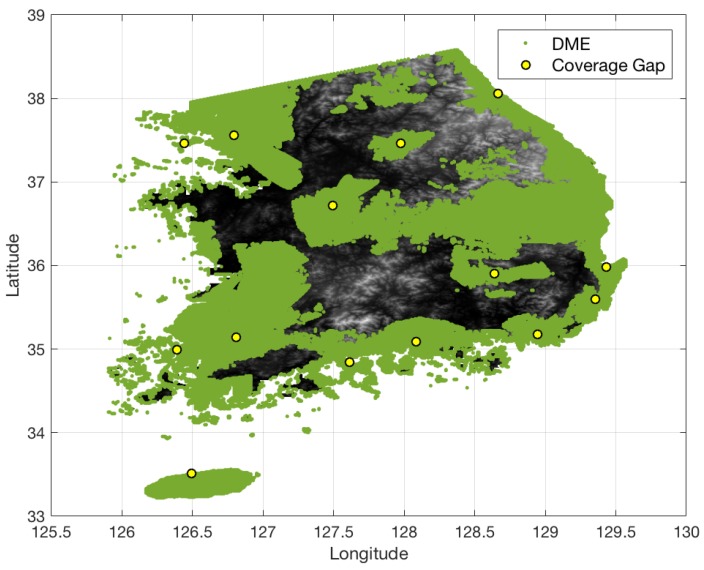
DME/DME coverage of 92.6 m horizontal positioning accuracy in South Korea with DME/S and the current DME ground network.

Figure 13 shows the terrain elevation profile in South Korea where elevation changes from 0 to 1800 m. The airspace in South Korea is similarly defined to that in South Florida, except that the minimum AGL altitude is 700 ft. Figure 14 shows the coverage gaps of 1.0 nm positioning accuracy with the current ground network of the legacy DME in South Korea. Unlike the South Florida region, there are significant coverage gaps even for 1.0 nm horizontal positioning accuracy. Figure 15 shows the horizontal positioning accuracy coverage gaps of 0.3 nm using DME/S and the current DME ground network, and Figure 16 shows an augmented network with DME/S to achieve 0.3 nm horizontal positioning accuracy, which has a total 38 sites including all of 14 existing DME sites and 24 new sites. Figure 17 shows the coverage gap of 92.6 m with DME/S and the current ground DME network, which is much larger than the 0.3 nm coverage gap. Because a significant number of the ground network augmentations are required even for 0.3 nm positioning accuracy, it seems unfeasible to realize DME/DME positioning that provides 92.6 m positioning accuracy in the entire region. Thus, no augmented ground network for 92.6 m horizontal positioning accuracy was sought. 

## 6. Discussion

The coverage analysis and augmented network placement shed light on which types of DME/DME operation is appropriate with legacy DME or DME/S. In the South Florida region, a DME/DME horizontal positioning accuracy of 1.0 nm is currently provided in most areas with legacy DME performance. However, Figure 8 indicated that non-minimal DME augmentation was needed to provide 0.3 nm horizontal positioning accuracy with legacy DME in this region. If DME/S is used, a ground network in Figure 12 providing 92.6 m horizontal positioning accuracy seems to be more appropriate than the ground network for 0.3 nm horizontal positioning accuracy shown in Figure 10, because one additional new DME site is needed to enable 92.6 m horizontal positioning accuracy, which is sufficient to replace current radar-based aircraft surveillance systems such as SSR.

**Figure 18:**
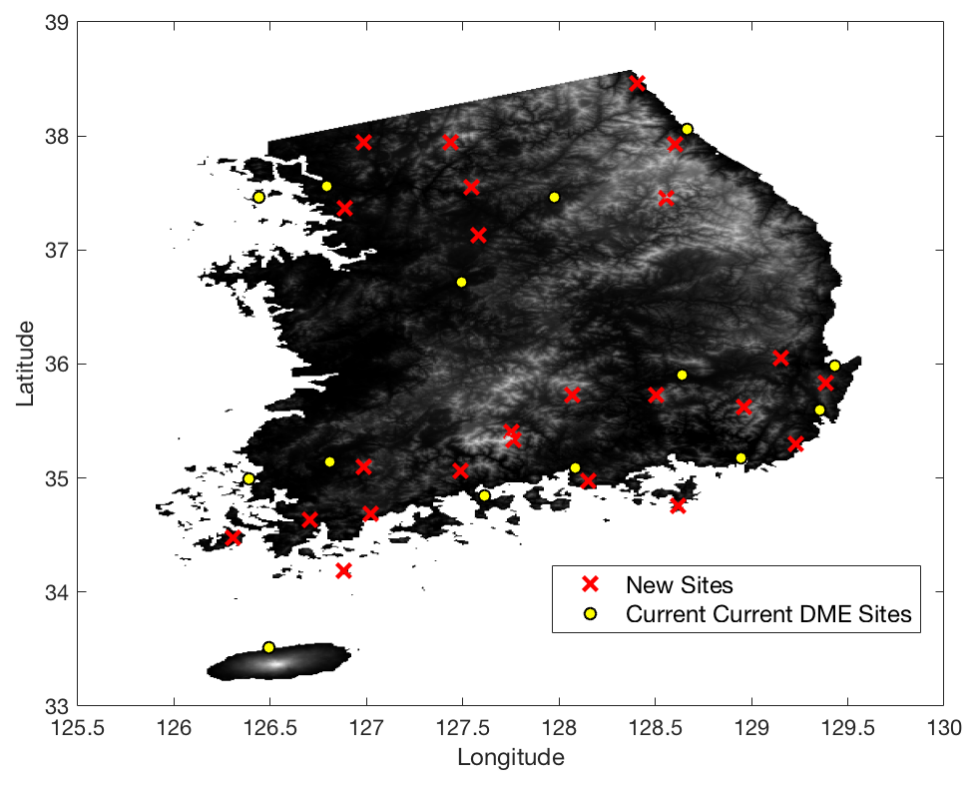
Augmented DME network for RNAV 0.3 nm with DME/S (five-channel scanning DME) in South Korea.

**Figure 19:**
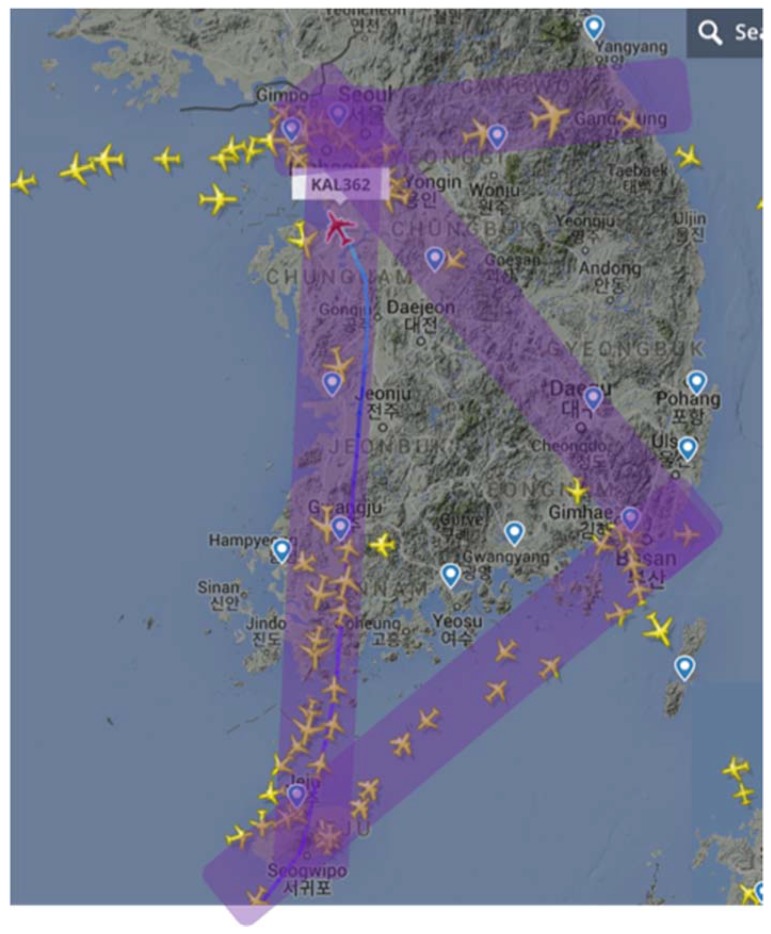
Heavy air traffic routes in South Korea are represented as shaded regions.

In South Korea, the difficult terrain condition makes it hard to achieve a 1.0 nm DME/DME horizontal positioning accuracy with the legacy DME performance. Most coverage gaps exist in low-altitude regions which have a limited line of sight to DMEs. The difficult terrain conditions also require a significant augmentation of the ground network, even with DME/S, to achieve 0.3 nm horizontal positioning accuracy. The positioning gap of 92.6 m is much larger than that of 0.3 nm, as shown in Figure 17; therefore, the appropriate positioning accuracy of alternative DME/DME would be 0.3 nm or less. To relax a required ground network augmentation in South Korea, a scanning DME of five channels can be used instead of three-channel scanning DME, since two additional ranging sources would help to lower *HDOP* values. Figure 18 shows the augmented ground DME network for 0.3 nm horizontal positioning accuracy with five channels. The network consists of a total 32 networks, with 11 current sites and 21 new sites. This requires six fewer ground transponders than three-channel scanning DMEs. Another way to relax the ground network augmentation is to allocate a DME/DME service area by reflecting heavy air traffic regions as shown in Figure 19. For example, an augmented DME network can be placed to provide 0.3 nm positioning accuracy in the shaded airspace only, which will be further investigated in the author’s future research.

## Figures and Tables

**Table 1:** Ranging accuracy budget of legacy DME, modern DME, and DME/SFOL.

95% Accuracy (m)	Legacy DME (DME Spec.)	Modern State-of-the-Art DME Performance [2,13]	DME/S
*Signal in Space*			
Reply Delay (m)	75.0	15.0	10.0 (DME/P)
	(FAA-E-2996)	(measured)	
Propagation to Aircraft/Noise (m)	127.3	39.0	16.8 (analysis)
	(derived)	(analysis)	
*Air/Avionics*			
Avionics Bias (m)	315.0	62.0	15.0 (DME/P)
	(DO-189)	(derived)	
Propagation to Transponder/Noise (m)	127.3	54	16.8 (analysis)
	(derived)	(analysis)	
Total root sum square (RSS) (m)	370.4	92.1	29.8
